# Supplementary material for: Causal Relationship between Aging and Anorexia Nervosa: A White-Matter-Microstructure-Mediated Mendelian Randomization Analysis
Source: Biomedicines. 2024 Aug 16;12(8):1874. doi: 10.3390/biomedicines12081874 (PMC11351342; doi:10.3390/biomedicines12081874)
Supplement: Supplementary file 1 [file biomedicines-12-01874-s001.zip › Supplementary Figure/Figure S8/Leaveoneout plot of MR analysis for TL to ubm-b-1523.pdf]

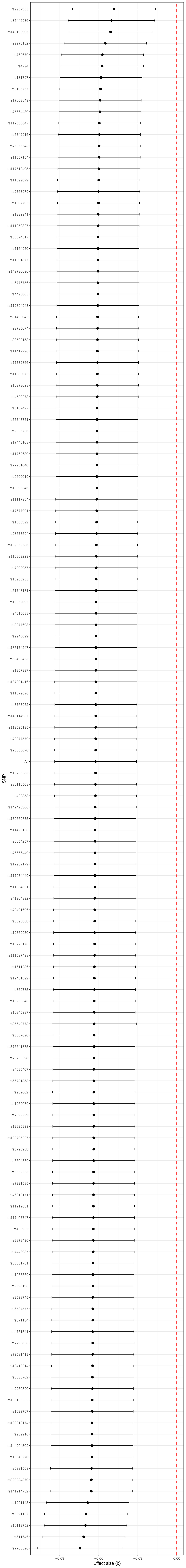
rs2967355
rs35446936
rs143190905
rs2276182
rs762679
rs4724
rs131797
rs8105767
rs17803849
rs75664430
rs117630647
rs5742915
rs76065543
rs11557154
rs117512405
rs11699829
rs2763979
rs1907702
rs1332941
rs111950327
rs80324517
rs7164950
rs11991877
rs142730696
rs6776756
rs4498805
rs112394943
rs61405042
rs3785074
rs28502153
rs11412296
rs77732866
rs11085072
rs16978028
rs4530278
rs8102497
rs55747751
rs2056726
rs17445108
rs11769630
rs77231040
rs9600019
rs10805346
rs11117354
rs17677991
rs1003322
rs28577594
rs182059586
rs116863223
rs7209057
rs10905255
rs61748181
rs13062095
rs4616688
rs2977608
rs9940099
rs185174247
rs59409453
rs1957937
rs137901416
rs11579626
rs3767952
rs145114957
rs113525195
rs79977579
rs28363070
All
rs10768683
rs80116508
rs429358
rs142426306
rs139669835
rs11426156
rs6054257
rs76666449
rs12932179
rs117034449
rs11584821
rs41304832
rs78491606
rs3093888
rs12369950
rs10773176
rs111527438
rs1611236
rs12451892
rs869785
rs13230646
rs10845387
rs35640778
rs6007020
rs376641875
rs73730598
rs4695407
rs66731853
rs932002
rs41269079
rs7099229
rs12925933
rs139795227
rs6790988
rs45604339
rs6669563
rs7221585
rs76219171
rs11212631
rs117407747
rs450962
rs9878436
rs4743037
rs56061761
rs1985369
rs9398196
rs2538745
rs6587577
rs871134
rs4731541
rs7790856
rs73581419
rs12412214
rs6536702
rs2230590
rs150150565
rs1023767
rs188918174
rs939916
rs144204502
rs10840270
rs6881568
rs202034370
rs141214782
rs1291143
rs3891167
rs10112752
rs611646
rs7705526
SNP
−0.09
−0.06
−0.03
0.00
Effect size (b)
